# Silence is deadly: a cluster-randomised controlled trial of a mental health help-seeking intervention for young men

**DOI:** 10.1186/s12889-017-4845-z

**Published:** 2017-10-23

**Authors:** Alison L. Calear, Michelle Banfield, Philip J. Batterham, Alyssa R. Morse, Owen Forbes, Bradley Carron-Arthur, Martin Fisk

**Affiliations:** 1 Centre for Mental Health Research, Research School of Population Health, The Australian National University, 63 Eggleston Road, Acton, ACT 2601 Australia; 2 Menslink, 27/27 Mulley Street, Holder, ACT 2611 Australia

**Keywords:** Help-seeking, Adolescent, Male, School-based intervention, Suicide, Mental health

## Abstract

**Background:**

Young men are consistently less likely to seek help for mental health problems than their female peers. This is particularly concerning given the high rates of suicide among male adolescents. The school system has been identified as an ideal setting for the implementation of prevention and early intervention programs for young people. The current trial aims to determine the effectiveness of the Silence is Deadly program in increasing positive help-seeking intentions for mental health problems and suicide among male secondary school students.

**Methods:**

This study is a two-arm, cluster-randomised, controlled trial that will compare the Silence is Deadly program to a wait-list control condition. Eight Australian high schools will be recruited to the trial, with male students in grades 11 and 12 (16 to 18 years of age) targeted for participation. The program is an innovative male-tailored suicide prevention intervention, comprising a presentation that emphasises role-modelling and legitimises help-seeking for personal and emotional problems, and a brief video that features celebrity athletes who counter existing male norms around help-seeking and encourage communication about personal and emotional issues. The program also includes a discussion of how to help a friend in distress and ends with a question and answer session. The primary outcome measure for the current study is help-seeking intentions. Secondary outcomes include help-seeking behaviour, help-seeking attitudes, help-seeking stigma, mental health symptoms, and suicidal ideation. Data will be collected pre-intervention, post-intervention, and at 3-month follow-up. Primary analyses will compare changes in help-seeking intentions for the intervention condition relative to the wait-list control condition using mixed-effects repeated-measures analyses that account for clustering within schools.

**Discussion:**

If proven to be effective, this targeted help-seeking intervention for adolescent males, which is currently only delivered in one jurisdiction, could be more widely delivered in Australian high schools. The Silence is Deadly program has the potential to significantly contribute to the mental health of young men in Australia by improving help-seeking for suicidality and mental health problems, allowing this population to better access treatment and support sooner.

**Trial registration:**

Australian New Zealand Clinical Trials Registry, ACTRN12617000658314. Registered on 8 May 2017.

**Electronic supplementary material:**

The online version of this article (10.1186/s12889-017-4845-z) contains supplementary material, which is available to authorized users.

## Background

### Young men have low rates of help seeking and are at high risk for suicide

Men are consistently less likely to seek help for mental health problems than women at all ages [1]. This difference is largest during adolescence; with only 13% of males with a mental health problem seeking professional help compared to 31% of females aged 16 to 24 years [1]. This is particularly concerning given that rates of suicide are three times higher among young men than young women [2], and that suicide is the most frequent cause of death in this age group [2]. Increasing rates of help seeking is an important public health target that could assist in lowering the rate of suicide among young men and reduce the burden of personal and emotional issues that may otherwise go unnoticed and untreated.

### Gender specific attitudes to help-seeking must be addressed

It is increasingly recognised that in order to improve rates of help-seeking among men, public health interventions must address the underlying attitudes and norms held by men that predicate planned behaviour [3, 4]. Young men are known to hold more negative attitudes towards help-seeking for mental health issues than young women [5], due in part to lower levels of openness to acknowledging psychological problems [6]. These differences in psychological openness are thought to be due to subjective masculine norms that encourage emotion suppression and lead to the stigmatisation of help-seeking for emotional problems [7].

This account is strengthened by research finding that up to 59% of the variance in mental health help-seeking attitudes among men can be explained by stigma and gender role conflict [8]. Further, a recent large all-male cohort study found that conformity to masculine norms around self-reliance and help-seeking was a significant predictor of increased risk of suicidal thinking [9]. Thus in order to change help-seeking behaviours, these norms and attitudes must be addressed. A recent systematic review of help-seeking interventions for depression, anxiety and psychological distress found promising evidence for the effectiveness of mental health literacy interventions in changing attitudes towards help-seeking (*d* = .12 to .53), particularly in young people [10]. However, none of the studies identified in this review analysed results according to gender and none were tailored to address the gender-based attitudes and perceived norms thought to drive male help-seeking behaviour.

### Tailoring shows promise but broader applications are needed

Research investigating tailored interventions to promote mental health help-seeking among young men has largely focused on two areas. Several studies have focused on determining the efficacy of different psychoeducational interventions by varying certain terms in mental health service brochures to be male friendly (e.g. [11–13]). Research has also compared men’s evaluations of, and preferences for, written or video examples of standard therapeutic interventions with interventions that use male-tailored language (e.g. [8, 14–17]). For example, substituting terms that highlight emotional problems with more generic references to personal problems has led to improved attitudes towards seeking help from a counselling service [17]. No research to date though has evaluated an intervention that is tailored, beyond language, to also address gender specific norms and attitudes that have been shown to be a driver of male help-seeking behaviour.

### Silence is deadly: A young male tailored intervention

Silence is Deadly is a multicomponent intervention delivered in schools that consists of a one-hour presentation, supporting website, videos, informational handouts and wristbands to show support for the cause [18]. This program has been delivered in Australian Capital Territory (ACT) schools since 2013 by the organisation Menslink, which was established in 2002 [19]. Similar to other programs in this field, it employs a psychoeducational design in which key statistics about mental health are presented alongside the personal experiences of the presenters, to normalise the existence of mental health issues in the community. In addition to this, it also targets the issue of low help-seeking rates among men and explicitly addresses the masculine norms that may underlie this reluctance to seek help. The program is driven by male role modelling and social norming and is delivered in partnership with the local celebrity athletes. Combining role-modelling of positive attitudes to help-seeking, multimedia presentations, and audience interaction, the program aims to reduce stigma around mental health problems and promote help-seeking behaviour among the target group of adolescent males.

### Aims of the trial

#### Primary aim

The primary aim of the trial is to determine the effectiveness of the Silence is Deadly intervention for improving intentions to seek help for personal and emotional problems at post-intervention and 3-month follow-up in school-aged males.

#### Secondary aims

The secondary aims of the trial are:

1. To test the effectiveness of the Silence is Deadly program in (a) increasing actual help-seeking behaviour, (b) improving help-seeking attitudes, and (c) reducing help-seeking stigma at post-intervention and 3-month follow-up.
2. To investigate the extent to which the effect of the intervention is mediated or moderated by the attitudinal characteristics of participants, including gender role conflict and help-seeking stigma. This aim is driven by the program’s intention to be effective for males who conform strongly to gender role norms, such as emotion suppression, and who hold more stigmatising attitudes towards seeking help for emotional problems.
3. To explore (a) if there is a differential effect of the intervention based on pre-intervention levels of psychological distress or suicidal ideation, and (b) the impact of the program on self-reported psychological distress and suicidal ideation at post-intervention and 3-month follow-up.
4. To explore the impact of the intervention on (a) student confidence to provide support to their peers at post-intervention and 3-month follow-up, and (b) school culture towards seeking help for emotional and/or personal problems. The latter aim will be investigated through semi-structured interviews with key staff members.

#### Trial hypotheses

- H1: The Silence is Deadly program will improve participants’ intentions to seek help for personal and emotional problems at post-intervention and 3-month follow-up, regardless of indicated levels of mental health symptoms or suicidality, relative to participants in the wait-list control condition.
- H2: The Silence is Deadly program will increase participants’ rates of informal and formal help-seeking for personal and emotional problems at 3-month follow-up relative to participants in the wait-list control condition.
- H3: The Silence is Deadly program will improve participants’ help-seeking attitudes and reduce help-seeking stigma at post-intervention and 3-month follow-up relative to participants in the wait-list control condition.
- H4: The Silence is Deadly program will be significantly more effective among participants who hold strong masculine role norms or higher help-seeking stigma at pre-intervention.

## Methods/design

### Study design

A two-arm cluster randomised controlled trial (RCT) will be conducted with one active intervention group and one wait-list control group. Surveys will be delivered on three measurement occasions - one-week pre-intervention, one-week post-intervention and at approximately 3-months follow-up. A qualitative evaluation of the intervention will also be conducted, comprising semi-structured interviews with selected staff members from all participating schools.

The ethical aspects of this study have been approved by The Australian National University Human Research Ethics Committee (protocol number 2017/004), Catholic Education Office of the Archdiocese of Canberra and Goulburn, and the ACT Education Directorate (reference RES-1719).

### Recruitment

At least 8 ACT schools, totalling approximately 800 students (100 male students per school), will be recruited to the trial via pre-existing relationships with the intervention provider Menslink. Due to Menslink’s longstanding involvement in the ACT community, providing services to school-aged males, the organisation has existing relationships with almost all schools in the region that enrol male students.

The research trial will be advertised via invitational letters and direct contact from Menslink staff to ACT schools with male students in grades 11 and 12 (16–18 years). Information and consent forms will be distributed to all participants prior to the completion of the pre-intervention survey, with participants aged 16 years and older being able to provide informed consent as a ‘mature minor’ [20]. If an insufficient number of participants can be drawn from this age group (16–18 years) then the sample will be extended to include males in Years 7 to 10 (12–16 years), and parental consent will be collected in addition to participant assent. All male students in participating schools could potentially attend the Silence is Deadly program, but only consenting students will be invited to complete the trial surveys.

Up to three staff members per school will be invited to participate in a semi-structured interview. The staff members targeted for this aspect of the research will be those holding key leadership and student wellbeing positions, such as year group coordinators, school psychologists and deputy principals.

### Randomisation

Each school will be cluster-randomised into either the intervention or wait-list control condition. This approach will reduce school and administrative burden and prevent contamination of the wait-list control condition. Randomisation will be conducted by a statistician who is not involved in the day-to-day management of the trial according to ICH Guidelines [21]. Given that entire schools will be randomised, a minimisation approach to randomisation will be taken in order to balance participant characteristics and numbers across conditions [22, 23]. Two factors will be used in the minimisation process: school type (private vs. public) and the number of students in grades 11 and 12 (<300 vs. 300+).

### Procedure

Prior to the commencement of the project in each school, trial managers will liaise with schools to introduce the project and discuss clinical protocols. These protocols will include the processes that will be followed if a student reports suicidality in the trial surveys. Student surveys will be screened by the trial managers following their completion to identify ‘at-risk’ respondents. School psychologists and/or another appropriate staff member at each school will be notified of ‘at-risk’ students, who will then be followed up in accordance with usual school procedures. Students will also be provided with help-seeking contacts (e.g., school psychologist/counsellor or general practitioner) and information (e.g., telephone helplines or websites). Additionally, Menslink is a provider of free counselling services to young men aged 12 to 25 years. Priority counselling appointments with Menslink will be offered to any participant who experiences distress or wishes to seek help as a result of completing the trial surveys or intervention.

At the commencement of the study, all consenting students in participating schools (both intervention and control) will be invited to complete a pre-intervention survey. Survey completion will be co-ordinated by the trial managers to ensure standardisation of survey delivery and student privacy. Surveys will be delivered during school time and will be completed online or on paper, depending on school preference and resourcing. The assessments will take approximately 15 to 30 min to complete and have been designed to reduce participant burden. All data will be de-identified and securely stored at The Australian National University, with access to the data restricted to trial personnel and investigators.

**Fig. 1:**
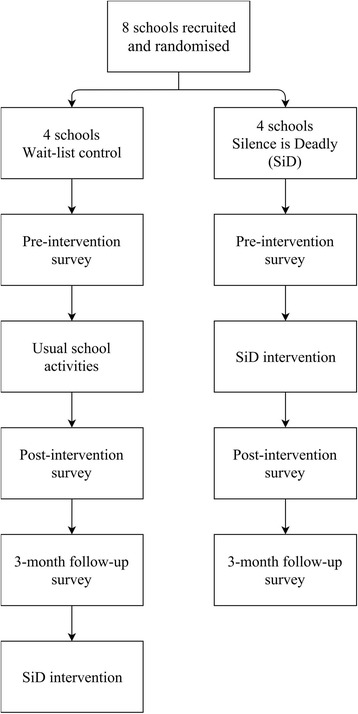
Flow diagram of trial progression

Following completion of the pre-intervention survey, students in intervention condition schools will receive the Silence is Deadly intervention during school hours. Students in control condition schools will undertake usual school activities during this period. Approximately 2 weeks after the pre-intervention survey, students in both intervention and control condition schools will complete the post-intervention survey. A final 3-month follow-up survey will also be administered to all students, after which time schools in the wait-list control condition will receive the Silence is Deadly program. Figure 1 presents the flow of participants in the trial (See Additional file 1: Attachment A for SPIRIT Checklist). Semi-structured interviews will also be conducted with selected staff members in all schools to qualitatively assess cultural changes in the school and attitudinal changes in students, associated with the Silence is Deadly program.

### Intervention

Silence is Deadly is a face-to-face multicomponent intervention targeted to males in secondary schools (12–18 years) in the ACT. The program is delivered to students over a 45–60 min group session by a facilitator, with traditional male role models (e.g., rugby players, military personnel). Program facilitators play a central role in the program, speaking about their own positive help-seeking experiences in response to personal difficulties, and the lessons they have learnt in countering traditional masculine norms. Professional athletes are another important element in the intervention, acting as role models in the delivery of the program and/or being included in a video that counters existing male norms that prevent positive help-seeking attitudes, and promote the importance of communicating about personal issues. All discussions are tailored to the male audience and include seeking support for mental health issues resulting from bullying, injuries, relationship issues and other circumstantial stressors, as well as how to ‘help a mate’ (i.e., start a conversation with a friend) who is not doing well. The program concludes with a question and answer session that lasts 5 to 15 min. The purpose of the intervention is to normalise the occurrence of mental health issues in young men and alter their attitudes to help-seeking by reframing them in the context of broader help-seeking norms for personal problems, which are less stigmatising and more compatible with typical gender roles.

### Assessments

Table 1 presents the scales that will be administered at each measurement occasion in the Silence is Deadly trial. With the exception of the Confidence Supporting Peers scale (see below), all measures have previously been evaluated with adolescent samples and have good psychometric properties.

**Table 1:** Measures for the silence is deadly trial

	Pre-intervention	Post-intervention	3-month follow-up	No. of items
Demographics	✔	✔	✔	7
Adapted GHSQ	✔	✔	✔	20
Adapted AHSQ	✔	✔	✔	22
Help-seeking from adults	✔	✔	✔	4
ATSPPH-SF	✔	✔	✔	10
SSOSH	✔	✔	✔	10
Confidence in supporting a friend	✔	✔	✔	4
DQ5	✔	✔	✔	5
YRBS	✔	✔	✔	1
GRCS-A	✔			9
Program evaluation^a^		✔		4

*Notes. AHSQ* Actual Help-Seeking Questionnaire, *ATSPPH-SF* Attitudes Toward Seeking Professional Psychological Help Scale - Short Form, *DQ5* Distress Questionnaire-5, *GHSQ* General Help-Seeking Questionnaire, *GRCS-A* Gender Role Conflict Scale—Adolescent Version, *SSOSH* Self-Stigma of Seeking Help Scale, *YRBS* Youth Risk Behaviour Survey

^a^Only students in intervention schools respond to these questions

#### Help-seeking intentions

An adapted version of the General Help-Seeking Questionnaire (GHSQ) [24] will be used in the current study to assess the primary outcome of help-seeking intentions for personal or emotional problems from 11 different formal and informal sources (e.g., friend, parent, psychologist, or teacher). Respondents indicate how likely they are to seek help from each of the sources on a scale ranging from 1 (extremely unlikely) to 7 (extremely likely). The wording of questions was adapted from ‘seeking help’ to ‘seeking advice’ to reflect the language used in the Silence is Deadly program.

#### Attitudes to seeking support from trusted adults

The 4-item Attitudes to Seeking Support from Trusted Adults scale will be used in the current study to assess help-seeking attitudes. Respondents indicate their agreement with items on a 4-point scale ranging from 1 (strongly disagree) to 4 (strongly agree). The scale is drawn from a US trial of the Sources of Strength suicide prevention program [25]. This scale also assesses respondents’ view of their family and friends’ norms regarding seeking help and support from trusted adults. Total scale scores on this scale can range from 4 to 16, with higher scores indicative of more positive help-seeking attitudes.

#### Attitudes to help-seeking from professionals

The Attitudes Toward Seeking Psychological Professional Help: Shortened Form (ATSPPH-SF) [26, 27] will be also be used to measure help-seeking attitudes. This scale aims to assess respondents’ attitudes towards self-reliance and professional support for personal and emotional problems. The ATSPPH-SF consists of 10 items: responses are on a 4-point scale ranging from 0 (disagree) to 3 (agree). Some items are negatively-worded to address positive response bias. After reverse-scoring of these items, total scale scores can range from 0 to 30, with higher scores reflecting more positive attitudes towards seeking help from professionals.

#### Help-seeking Behaviours

The adapted Actual Help-Seeking Questionnaire (AHSQ) [24], will be used to assess recent actual help-seeking behaviour for personal or emotional problems and assesses the same 11 sources of help as the GHSQ. Respondents are asked whether or not they have sought help for personal or emotional problems from 11 different formal and informal sources (e.g., friend, parent, psychologist, or teacher) in the past 3 months. The question wording was adapted from ‘seeking help’ to ‘seeking advice’ to reflect the language used in the Silence is Deadly program.

#### Stigma of help-seeking

The Self-Stigma of Seeking Help Scale (SSOSH) [28] will be used in the current trial to assess the extent to which respondents hold stigmatised attitudes towards seeking or receiving support for their own personal or emotional problems. Attitudes are measured by 10 items on a 5-point scale ranging from 1 (strongly disagree) to 5 (strongly agree). Some items are negatively-worded to address positive response bias. After reverse-scoring of these items, total scale can range from 10 to 50, with higher scores reflecting more self-stigma towards seeking help.

#### Mental health symptoms

General psychological distress will be assessed with the Distress Questionnaire Scale – 5 (DQ5) [29]. This scale consists of 5 items rated on a 5-point scale from 1 (never) to 5 (always), with total scale scores ranging from 5 to 25. Higher scores on the DQ-5 reflect greater psychological distress.

#### Suicidality

One item from the Youth Risk Behaviour Survey (YRBS) [30, 31] will be administered to assess if the respondent has had suicidal ideation during the past 12 months (response options: yes or no). Research indicates that the measurement of suicidal behaviours in adolescents is acceptable and does not pose an iatrogenic risk [32].

#### Gender role conflict

The Restrictive Emotionality subscale of the Gender Role Conflict Scale - Adolescent Version (GRCS-A) [33] will be used to assess conformity to gender norms. The sub-scale contains 9 items that are rated on a 6-point scale ranging from 1 (strongly disagree) to 6 (strongly agree). Total scale scores can range from 9 to 54, with higher score indicative of greater conformity to gender norms regarding emotional restriction.

#### Confidence supporting peers

Four items, adapted from literature evaluating the Mental Health First Aid (MHFA) training program [34], will be used to assess respondents’ confidence and willingness to support a friend or peer who is experiencing personal difficulties. Responses are on a 5-point scale from 1 (not at all) to 5 (extremely) or a Yes/No/Don’t know scale.

In addition to the measures above, participants in intervention schools will be asked to complete a number of open-ended evaluation questions at post-intervention. These qualitative data will provide insight into strengths and areas of improvement for the Silence is Deadly program.

### Sample size and power calculations

A sample of 800 adolescents from 8 high schools (100 students per school) will be targeted for recruitment to the current study. Calculation of the required sample size was based on detecting a small-moderate post-intervention effect size of f = 0.2. Assuming correlation between repeated measures of 0.5, intra-class correlation of 0.03 (accounting for clustering within schools) and 20% attrition, the trial will have >85% power to find a small difference (Cohens f = 0.2) between groups.

### Statistical analysis

Analyses of primary and secondary effectiveness outcomes will be conducted using a mixed-model repeated measures (MMRM) approach [35], the standard and most robust methodology for analysing randomised controlled trials. This approach is able to include participants with missing data without using biased imputation techniques, assuming that data are missing-at-random. Further, by incorporating appropriate random effects for each school, MMRM can accommodate and assess the strength and significance of clustering effects. Identification of moderating factors, such as mental health symptoms, suicide behaviours, stigma and gender-role conflict, will be conducted by introducing three-way interaction terms of time × condition × moderator, also using MMRM. Analysis of binary outcomes will be undertaken using binary mixed models, analogous to linear MMRM.

Qualitative analysis of the semi-structured interviews will be conducted using Framework Analysis. Framework Analysis was designed for addressing social policy research questions [36] and has been used frequently in health research [37]. It follows a systematic process of inductive coding, generating a table of findings across participants that is conducive to interpretation and input from multiple researchers.

## Discussion

The need for effective and tailored interventions for personal and emotional problems in males is high, particularly given the rates of suicide and mental health difficulties observed in this population [1, 2]. The current project represents an opportunity to evaluate a program specifically designed for males, which primarily aims to increase help-seeking intentions for personal and emotional problems. The targeting of help-seeking intentions, and help-seeking attitudes and behaviours as secondary outcomes, within this program is important given the low rates of help-seeking evident in this population for mental health problems and suicide [5–8].

The intervention tested in this trial is also novel in its approach to targeting male gender-specific attitudes and obstacles that drive poor help-seeking intentions, attitudes and behaviours in this population. If found to be effective, it could provide a key solution to promoting help-seeking in young males, which is the cornerstone to preventing and reducing mental health problems, disengagement and suicide risk. Mental health and suicide prevention systems are lacking in Australian schools and the program tested in this trial has the potential to significantly impact the help-seeking intentions, attitudes and behaviours of young Australian men and stimulate more high quality research in this critical area.

Results of the study will be disseminated in peer-reviewed articles, conference presentations and in policy/practice forums relevant to schools, with a summary of findings posted on the website of the Centre for Mental Health Research for the information of participating schools, students and other stakeholders.

### Trial status

Participants are currently being recruited to the trial.

## Additional file

Additional file 1: Attachment A for SPIRIT Checklist. (DOC 121 kb)

## Abbreviations

ACT: Australian Capital Territory
AHSQ: Actual Help-Seeking Questionnaire
ATSPPH-SF: Attitudes Toward Seeking Psychological Professional Help: Shortened Form
DQ5: Distress Questionnaire Scale – 5
GHSQ: General Help-Seeking Questionnaire
GRCS-A: Gender Role Conflict Scale - Adolescent Version
MHFA: Mental Health First Aid
MMRM: Mixed-model repeated measures
SSOSH: Self-Stigma of Seeking Help Scale
YRBS: Youth Risk Behaviour Survey
